# Small Cell Carcinoma of the Rectum Associated With a Tubulovillous Adenoma: An Atypical Case Presentation

**DOI:** 10.7759/cureus.55995

**Published:** 2024-03-11

**Authors:** Mena Louis, Elaine Lelli, Fernando Aycinena, Ezra Ellis

**Affiliations:** 1 General Surgery, Northeast Georgia Medical Center Gainsville, Gainesville , USA; 2 Surgery, Northeast Georgia Medical Center Braselton, Georgia, USA; 3 Colorectal Surgery, Northeast Georgia Medical Center Gainesville, Gainesville , USA; 4 Pathology, Northeast Georgia Medical Center Gainesville, Gainesville, USA

**Keywords:** advanced endoscopic techniques, multidisciplinary cancer treatment, robotic transanal surgery, hepatocellular carcinoma, neuroendocrine tumor rectum, tubulovillous adenoma, small cell carcinoma of the rectum

## Abstract

Small cell carcinoma of the rectum (SCCR) is a rare and aggressive neuroendocrine tumor. Its association with a tubulovillous adenoma is an exceptional occurrence, presenting significant implications for diagnosis and treatment. This case report details a 62-year-old male, undergoing treatment for hepatocellular carcinoma, presented with symptoms of diarrhea. A colonoscopy initially suggested a benign tubulovillous adenoma, but the presence of discordant clinical findings led to further evaluation. The final diagnosis, established post-surgery, was SCCR originating from a tubulovillous adenoma. This case highlights the diagnostic challenges when unusual presentations arise from atypical pathological findings, especially in patients with concurrent malignancies. The management followed standard care protocols, including robotic transanal surgery, despite the patient's ongoing HCC treatment. This case adds to the limited existing literature on SCCR, particularly its rare association with a tubulovillous adenoma. It emphasizes the importance of a multi-disciplinary approach in diagnosing and managing rare entities in colorectal cancer while demonstrating the feasibility of standard care in patients with complex comorbidities.

## Introduction

Neuroendocrine tumors (NETs) represent a heterogeneous group of malignancies originating from neuroendocrine cells throughout the body [1,2]. In the gastrointestinal tract, the incidence of NETs varies, accounting for approximately 0.1% to 3.9% of all colorectal malignancies [2-4]. Within this spectrum, small cell neuroendocrine carcinomas (SNECs) in the rectum are exceedingly rare and aggressive, often presenting diagnostic and therapeutic challenges [2,3]. The World Health Organization's classification, updated in 2019, subdivides neuroendocrine neoplasms into well-differentiated NETs (G1, G2, and G3) and poorly differentiated neuroendocrine carcinomas, which include small-cell and large-cell types [5]. The distinction is clinically significant as poorly differentiated variants, including SNEC, tend to follow a more aggressive course compared to their well-differentiated counterparts [5].

Concurrently, hepatocellular carcinoma (HCC) stands as a leading cause of cancer-related deaths worldwide, often associated with chronic liver diseases like the hepatitis C virus [6,7]. The management of HCC often involves complex therapeutic regimens, adding layers of complexity when other malignancies co-exist [8]. The occurrence of a second, unrelated malignancy in a patient already undergoing treatment for HCC is rare and raises questions about diagnostic accuracy, treatment prioritization, and overall prognosis [9,10]. This case report presents the unique co-occurrence of a rectal SNEC in a patient undergoing treatment for HCC.

Small cell carcinoma is a highly aggressive form of cancer most commonly associated with the lung [11]. When it occurs in the rectum, it constitutes a small fraction of colorectal malignancies [12]. The rare incidence of rectal small cell carcinoma presents unique challenges in diagnosis and management, making each case a subject of clinical interest [13,14].

While tubulovillous adenomas are widely recognized as precursors to colorectal adenocarcinomas, their progression to small-cell neuroendocrine tumors (NETs) is not well understood and appears to be exceedingly rare [15]. It is unclear whether the classic adenoma-carcinoma sequence typical of colorectal adenocarcinomas applies to the transition from adenoma to NET, or whether these conditions coexist independently within the same lesion [16]. This suggests the possibility of an alternative pathway of malignant transformation, which may involve the neuroendocrine cells within the adenomatous tissue [17]. Such ambiguity presents significant diagnostic challenges, as clinicians must be vigilant for signs of aggressive cancer even when encountering lesions that traditionally suggest a benign nature [15].

This case report aims to explore diagnosing and managing small-cell rectal carcinoma, particularly when it originates from a colorectal adenoma.

## Case presentation

A 62-year-old male, a former smoker with a known history of chronic hepatitis C virus (eradication therapy completed in 2014), and compensated liver cirrhosis, presented with a complex medical background. He was actively undergoing treatment for hepatocellular carcinoma (HCC) with Avastin and was also on anticoagulation therapy (Apixaban) for portal vein thrombosis. In addition, he had a history of esophageal varices without high-risk features, for which he had recently undergone band ligation.

**Figure 1:**
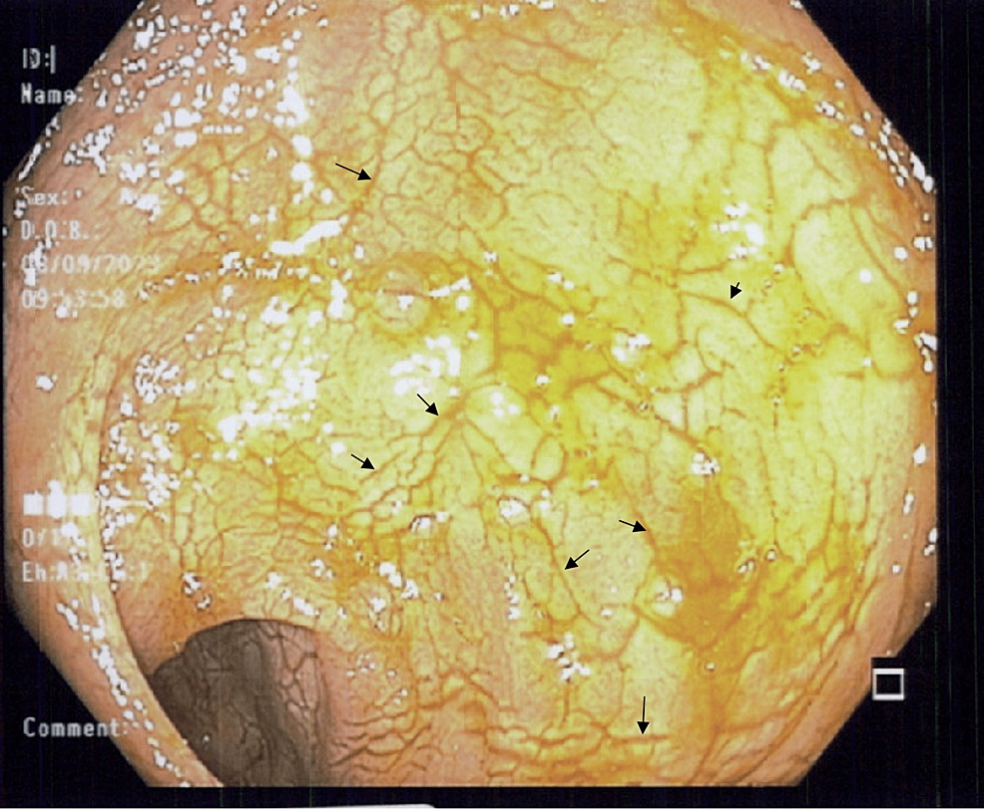
Colonoscopy picture of the rectum showing abnormal mucosa with circumferential mucosal cracking (black arrows).

**Figure 2:**
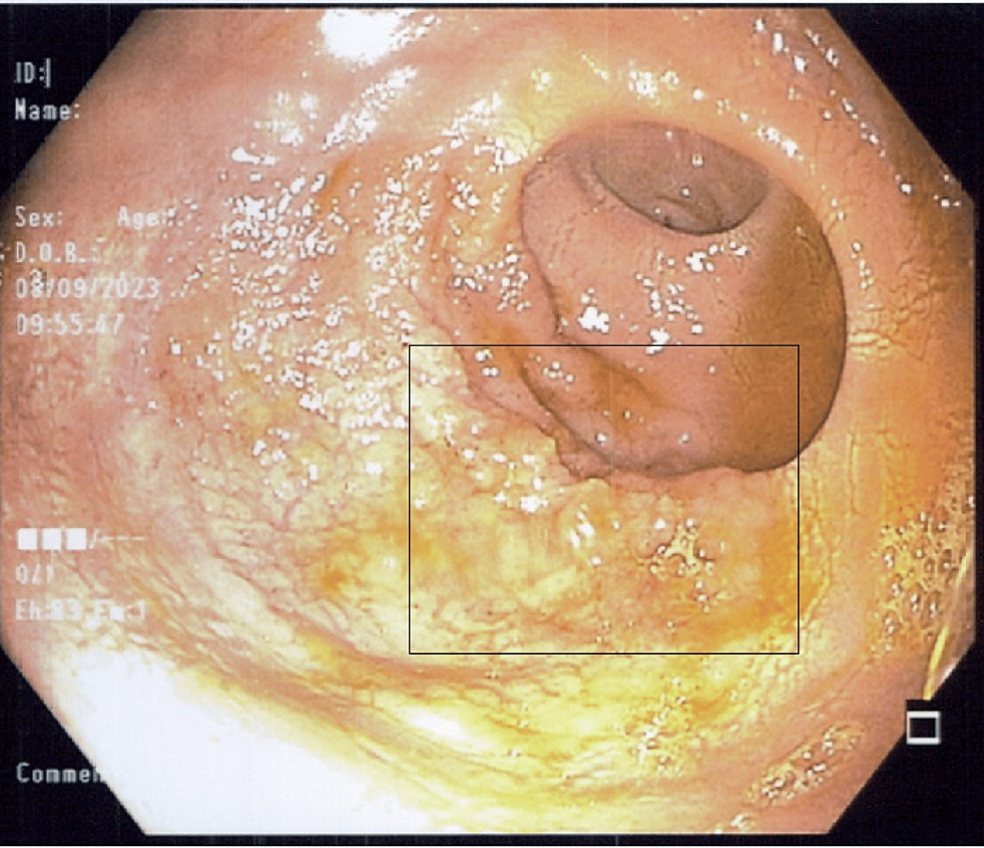
Colonoscopy picture of the rectum showing poorly defined 4 cm villous mass at the level of the first valve of Houston (black square).

The patient was initially referred for colonoscopy due to complaints of diarrhea occurring three to four times a day, rated as 5-6 on the Bristol stool scale. During the procedure, a laterally spreading hemi-circumferential mass was identified 5 cm from the anal verge, which was not amenable to endoscopic removal. Biopsies taken at that time were suggestive of a tubular adenoma without dysplasia. Following this, he was referred to colorectal surgery for further evaluation. Examination under anesthesia (EUA), flexible sigmoidoscopy, and intraoperative biopsies were performed one month later, revealing an abnormal mucosa with circumferential mucosal cracking (Figure 1). A poorly defined 4 cm villous mass was observed at the level of the first valve of Houston (Figure 2). Repeat biopsies were consistent with an advanced histology of a tubulovillous adenoma without dysplasia. 

**Figure 3:**
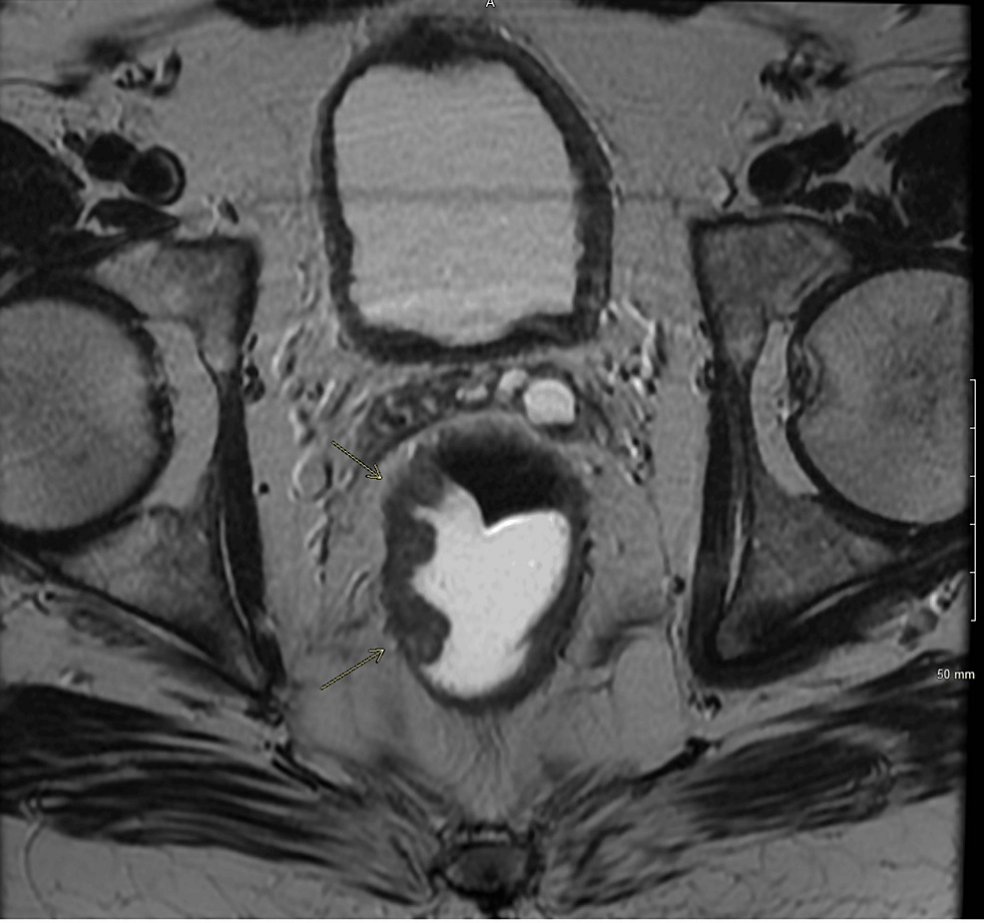
Axial MRI of the rectum with contrast revealing a 3.3 cm polypoid lesion centered in the mid-rectum. The lesion extends to less than 1 mm beyond the muscularis propria. The distance to mesorectal fascia (circumferential resection margin/CRM): 1.00 cm.

**Figure 4:**
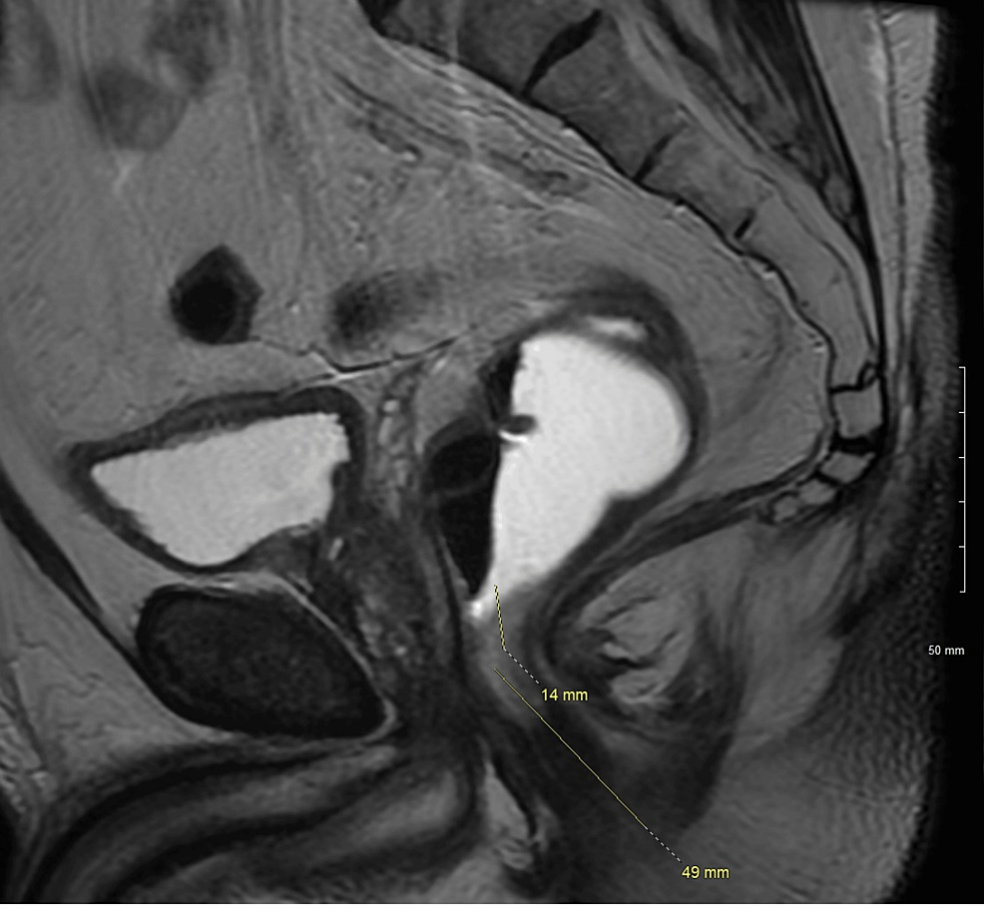
Sagittal MRI rectum with contrast showing the lowest extent of tumor distance from the anal verge: 6.5 cm. The lowest extent of the tumor is 1.4 cm from the top of the anal sphincter. Tumor: Less than 180 degrees of involvement of the rectal circumference, from 7 to 11 o'clock. The lesion is below the peritoneal reflection. No suspicious pelvic sidewall or extra mesorectal lymph nodes.

**Figure 5:**
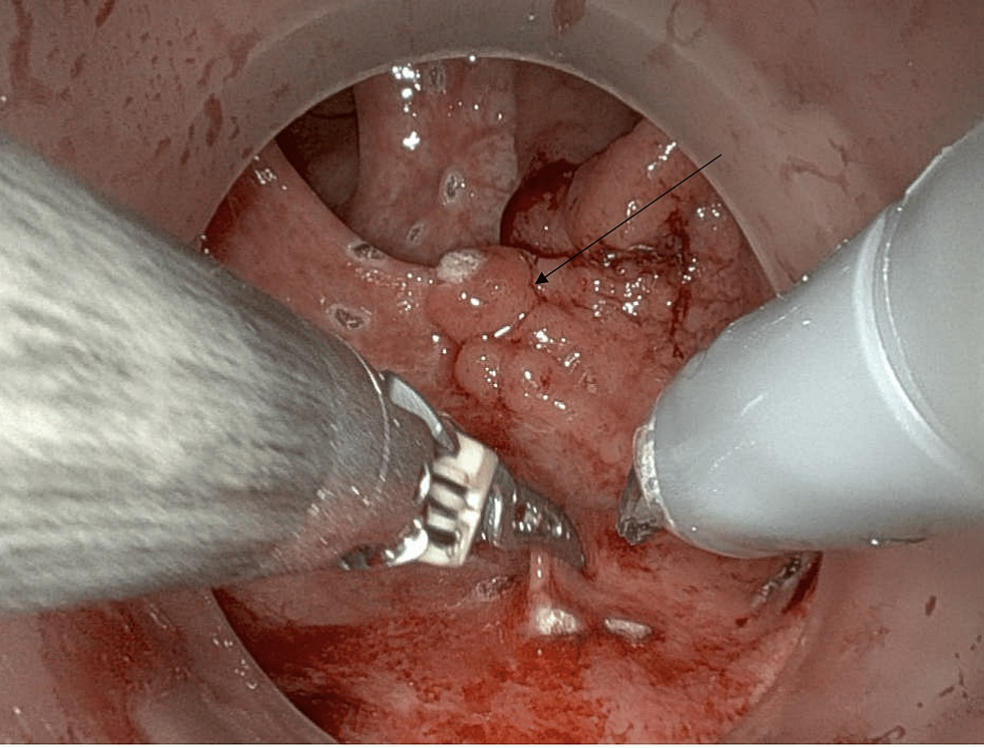
Intraoperative image during robotic trans-anal minimally invasive surgery (TAMIS) showing the rectal polyp pre-excision.

**Figure 6:**
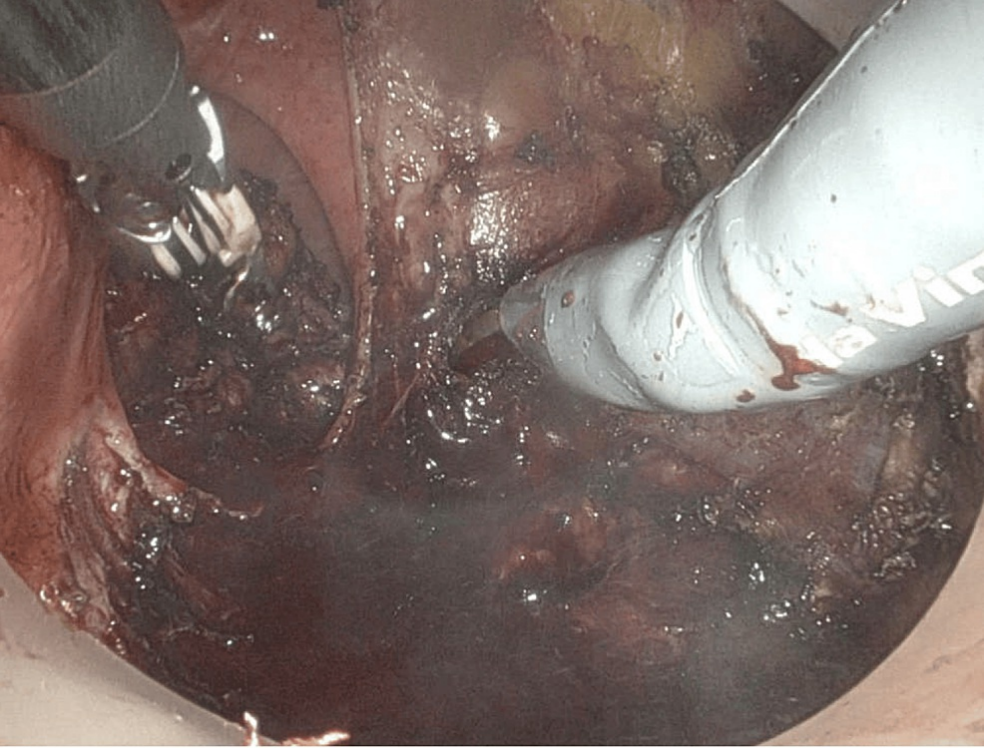
Intraoperative image during robotic trans-anal minimally invasive surgery (TAMIS) showing full-thickness local excision of the rectal polyp.

**Figure 7:**
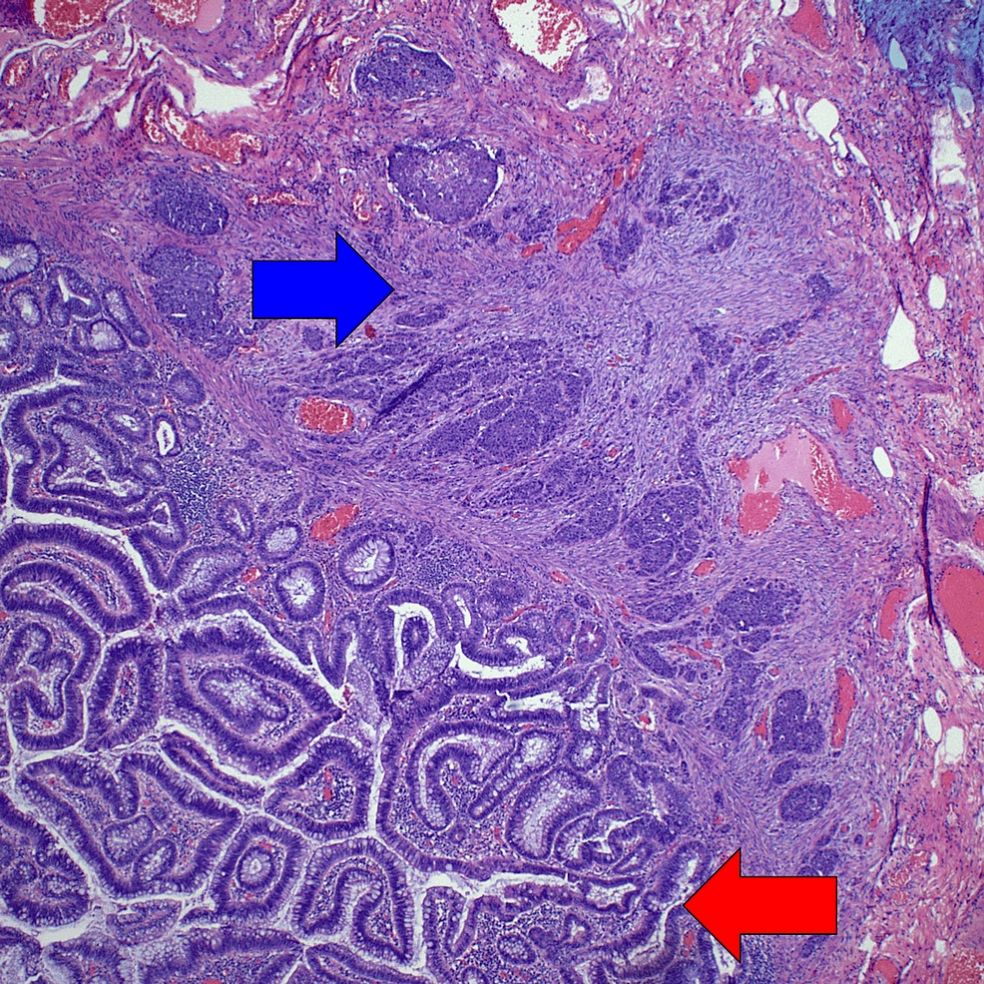
Tubulovillous adenoma (red arrow) and small cell carcinoma (blue arrow).

Given the discordant pathology findings and the need for precise surgical planning, a rectal MRI was performed to search for regional lymphadenopathy, as the findings were at least indicative of an advanced polyp. The MRI revealed a 3.3 cm polypoid lesion in the mid-rectum, 6 cm from the anal verge, raising suspicions of a mrT3aN0 stage lesion (Figures 3, 4). Given these findings, the patient underwent a robotic trans-anal minimally invasive surgery (TAMIS) procedure for definitive resection (Figures 5, 6). The pathology revealed a small cell carcinoma arising within a tubulovillous adenoma with high-grade dysplasia (Figure 7). Immunohistochemical staining confirmed lymphovascular invasion and was supportive of a small cell carcinoma diagnosis, with a Ki-67 index of 40%.

The patient experienced postoperative bleeding complications 24 hours after resuming anticoagulation and required a brief hospital stay for observation. He was subsequently discharged without further complications.

## Discussion

This case of small cell carcinoma of the rectum, arising in association with a tubulovillous adenoma, illustrates a rare occurrence in colorectal cancer pathology. The initial diagnosis of a benign adenoma through colonoscopy, followed by the unexpected finding of a malignant small cell carcinoma post-surgery, highlights the need for vigilance in cases with atypical presentations [9,14]. The presence of hepatocellular carcinoma (HCC) in this patient added a layer to the clinical picture, though it is important to note that the management of the rectal carcinoma was considered independent of the HCC. The standard protocols for colorectal cancer diagnosis and treatment were followed, including the use of robotic transanal surgery, which is an established method in such cases. This approach allowed for a definitive diagnosis and effective treatment without undue complexity, despite the patient's ongoing chemotherapy for HCC.

While HCC has been extensively studied and its epidemiological footprint is well understood, SNEC remains elusive in the medical literature, particularly when located in the rectum [13,18]. 

The initial diagnostic workup revealed discordant pathology, highlighting the limitations of biopsy-based diagnoses, the critical role of comprehensive imaging studies, and sound clinical judgment. In this case, given the discordant clinical, histological, and imaging findings, coupled with the patient's high-risk surgical comorbidities and his preference to avoid a colostomy, a 'total biopsy concept' via a transanal approach was deemed appropriate [19-22]. 

The management of this patient was intricate due to multiple comorbidities, including active chemotherapy, cirrhosis, and the need for anticoagulation for portal vein thrombosis. The choice of adjuvant therapy is also challenging; the high Ki-67 index of 40% and the confirmed lymphovascular invasion point toward an aggressive disease course, necessitating a consideration for chemotherapy despite the absence of distant metastases [23,24]. 

Guidelines for colorectal neuroendocrine tumors (NETs) larger than 2 cm advocate for aggressive surgical resection, which in this scenario would necessitate a proctectomy with mesorectal excision to ensure thorough lymphadenectomy [5]. However, this approach should be considered cautiously, as these recommendations encompass lesions with adverse risk factors, including lymphovascular invasion, yet do not explicitly distinguish between well-differentiated and poorly differentiated tumors, the latter of which exhibit more aggressive behavior.

This case was discussed at our multidisciplinary tumor board meeting, where the consensus emerged with a strong inclination towards systemic chemotherapy. Additionally, there was an agreement that the potential benefits of adjuvant radiation therapy should be thoroughly explored in further discussions.

While the association of small cell carcinoma with a tubulovillous adenoma is not common, it does not, in this case, represent a diagnostic or therapeutic dilemma. The clinical decisions made were in line with standard practices for colorectal malignancies, demonstrating that even in the presence of atypical pathological findings, established diagnostic and treatment pathways can be effectively utilized.

This case serves as a significant addition to the scant literature on rectal SNEC and its management. It emphasizes the necessity of considering multiple, potentially unrelated severe diagnoses in patients with complicated medical histories. 

## Conclusions

This case report details a rare instance of small cell carcinoma of the rectum emerging from a tubulovillous adenoma, in a patient with hepatocellular carcinoma. The key takeaway from this case is the demonstration of how established clinical protocols can effectively manage even atypical presentations of colorectal cancer. Despite the unusual pathological findings, the treatment followed standard colorectal cancer management practices, including robotic transanal surgery.

While the coexistence of different malignancies in a single patient can present a complex clinical scenario, this case exemplifies that such complexities can be navigated successfully within the framework of standard medical care.
